# Non-Invasive Breath Analysis for Disease Screening and Diagnoses

**DOI:** 10.3390/bios12040235

**Published:** 2022-04-12

**Authors:** Hyun Jin Jung, Ronny Priefer

**Affiliations:** Department of Pharmaceutical Sciences, Massachusetts College of Pharmacy and Health Sciences University, Boston, MA 02115, USA; hjung1@stu.mcphs.edu

Lower respiratory infections are a deadly communicable disease ranked as the fourth leading cause of death globally, with nearly 2.6 million succumbing annually [1]. Acute lower respiratory tract infections in children have sadly been shown to increase the risk of developing a chronic respiratory disease later in life. Additionally, respiratory tract infections caused by influenza kill between 250,000 and 500,000 people globally and costs between USD 71 and 167 billion annually [2]. Early detection and accurate diagnosis of infectious disease are important to ultimately improve the effectiveness of treatments, determine the correct use of antibiotics, avoid long-term complications, and help prevent or stop an outbreak.

Many different laboratory tests are currently used to identify infectious microorganisms. These include tests on blood, urine, tissue, cerebrospinal fluid, stool, sputum, and mucus from the nose or throat, as well as fluid from the genital area [3]. These existing methods can also be divided into either invasive or non-invasive. Invasive methods include esophagogastroduodenoscopy (EGD), with collection for gastric biopsies and subsequent histological staining. It is often used to detect Helicobacter pylori infection in the gut. This is expensive, causes patient discomfort, and sometimes yields false negative results due to sampling errors. However, there are a growing number of non-invasive methods for detecting H. pylori infection, such as serological, fecal antigen, and ^13^C-urea breath testing (^13^C-UBT) [4,5].

Utilizing the breath to screen or diagnose diseases has become increasingly popular. Human-exhaled breath contains over 3000 volatile organic compounds (VOCs). Breath VOCs are produced by our own biologic processes, as well as by microorganisms as part of their metabolism. These can be detected and quantified utilizing various instrumentations, such as gas chromatography/mass spectrometry or eNose detection [6]. These and other advanced technologies specifically examine the volatile metabolic fingerprints generated by our body and its microorganism flora in order to discriminate between different aliments. Thus, VOCs are being evaluated extensively as potential biomarkers and as a non-invasive approach to analyze a multitude of diseases, such as diabetes, lactose intolerance, cancer, infections, [7,8,9] and/or lifestyle choices, such as keto-diet [10] or cannabis use [11]. For example, Gastric Emptying Breath Test (GEBT), H_2_ breath monitoring, and NIOX^®^ are FDA-approved tests [7,12,13]. GEBT is used to diagnose gastroparesis, H_2_ breath analysis is used for the diagnosis of lactose intolerance [9], and NIOX measures markers for airway inflammation. By identifying disease-specific biomarkers, early detection could prevent serious complications, lowering mortality rates and reducing the unnecessary use of medicines.

With the fantastic advances in technology, especially nanotechnology, the opportunity to use these in the realm of healthcare, specifically to detect small quantities of biomarkers, is an obvious next step. This Special Issue, “Non-invasive Medical Devices for Detection and Monitoring within Healthcare”, is designed not only to showcase what has already been accomplished to improve detection efficacy, but also the translation of these advancements into improving patient experiences and outcomes.

## Data Availability

Data sharing not applicable.
